# Supplementary material for: Does sex of the jockey influence racehorse physiology and performance
Source: PLoS One. 2022 Aug 31;17(8):e0273310. doi: 10.1371/journal.pone.0273310 (PMC9432741; doi:10.1371/journal.pone.0273310)
Supplement: Attachment — Submitted filename: Response to Reviewers.docx [file pone.0273310.s001.docx]

**Reviewer #1:** The authors are to be commended for their significant work in revising this manuscript. There are substantial changes that address most of the comments, with great improvement in scope and clarity. However, there are still few issues need to be noticed, especially in the introduction part. Some suggestions are listed in the specific comments below

Specific comments:

1. In the last paragraph of introduction part, please mention the aim of this study.

Page 4, lines 144-153. A re-written aim/hypothesis has been included, replacing the previous clearly stated hypothesis.

2. In the conclusion part, line 549-558, “We hope that the results of the study…to better quality mounts in racing events.” It is recommended to improve the logic of these sentences. Findings of this manuscript should be put before the implication and highlights.

Page 14, lines 542-553. A re-written concluding paragraph has been included

**Reviewer #2:** Most of the previous concerns were addressed in the revised manuscript. However, after further examination, there are still some small issues that need to be addressed before bringing this up to a publishable standard, please find them below.

1. Lines 54-55, 57, it is suggested that the authors could change it to “p > 0.05” since there is no significance.

Page 2, lines 54-55, 57. Revision made.

2. In terms of my previous comment “The results are duplicates in the results session, on the tables, and also in the discussion session. I recommend the authors keep just the table on the results table and make the discussion stronger. The text on the results session with the description of the table is not helping the reader.”, the results section describes the findings of this paper in considerable detail, while these details have already been included in the tables and figures. Therefore, the author may consider making some deletions while keeping the main results of this paper here.

Page 8 -, the authors are uncertain what specific changes are required here. The legends of each table and graph help the reader understand that data. In the results section, a summary description is given of each figure or table and the exact data is not repeated as either text/table/graph. Any results in the discussion are specifically incorporated there to help elucidate that discussion point, meaning the manuscript has more of a narrative flow. I do not think ‘making the discussion stronger’ is valid, as essentially any elements of a discussion are the authors subjective comment on their objectively acquired and reported results. I believe this is what is reported here.
